# Awareness of inflammatory bowel disease among the general population of Al-Baha region, Saudi Arabia

**DOI:** 10.25122/jml-2023-0463

**Published:** 2024-02

**Authors:** Turki Alkully, Sarah Taishan, Wafaa Taishan, Lara Alsakka, Njood Alghamdi, Nouf Alghamdi, Mohammed Alghamdi

**Affiliations:** 1 Gastroenterology and Advanced Endoscopy, Department of Internal Medicine, Faculty of Medicine, Al-Baha University, Saudi Arabia; 2 Faculty of Medicine, Al-Baha University, Al-Baha, Saudi Arabia

**Keywords:** inflammatory bowel disease, Saudi Arabia, Crohn’s disease, ulcerative colitis

## Abstract

Inflammatory bowel disease (IBD) is a chronic gastrointestinal disorder that encompasses Crohn’s disease (CD) and ulcerative colitis (UC). IBD can be debilitating and has severe effects on the quality of life of the affected individuals. However, despite the increasing frequency of IBD around the world, the general population lacks knowledge and comprehension of this illness. The aim of this study was to determine the level of knowledge and awareness of IBD among the general population in Al-Baha region, Saudi Arabia. We carried out a cross-sectional study using an online self-administered validated questionnaire. The questionnaire included demographic questions, as well as questions regarding knowledge and awareness of IBD. The study included 473 participants selected by convenience random sampling with equal chance of selection. Approximately 61% of the participants had never heard about IBD, and for those who did, social media was the most common source of information (40.6%). Also, the majority of participants had limited knowledge about the different types of IBD (74%), their symptoms, and long-term effects. Women had a significantly higher level of adequate knowledge (12.1%) compared to men (4.8%) (*P* = 0.011). The study demonstrated a substantial lack of understanding and awareness of IBD among the general population in Al-Baha region, notably regarding the different types of IBD, their symptoms, and their long-term effects. The study underscores the need for further educational initiatives and resources to raise public knowledge and comprehension of IBD globally.

## INTRODUCTION

Inflammatory bowel disease (IBD) is a chronic inflammatory condition in genetically susceptible individuals after an exaggerated immune response to a normal stimulus like food or flora [1]. IBD is prevalent in most countries and is becoming an emerging disease globally [2]; however, there are limited data on its epidemiology in the Middle East [3,4]. According to a cross-sectional observational study carried out in the western region of Saudi Arabia, the general population in Saudi Arabia has an unacceptable level of awareness of IBD. Women, young adults (aged 31–40 years), educated individuals (with a PhD), and those who had previously dealt with patients with IBD had better awareness compared to the rest of the population. National acts are essential to improve public awareness of the disease [5]. An online survey that examined public knowledge of IBD in Saudi Arabia between February and March 2023 found a low level of IBD awareness among the general population, supporting findings from other countries. Future research should identify effective educational interventions to increase public awareness of this group of diseases, which would ultimately facilitate early diagnosis and improve patient outcomes [6]. Another retrospective study conducted in Riyadh between 1970 and 2008 concluded that the incidence of IBD is increasing in Saudi Arabia [7]. A retrospective study carried out in Jeddah between January 2002 and July 2007 showed that 19.1% of 711 colon biopsies were diagnosed as IBD [8]. The prevalence of perceived stigma among adults with IBD may reach 84% [9], highlighting the need for increased community awareness of this chronic disease, as it can negatively impact treatment adherence, quality of life, and practical needs. The condition also affects the educational and occupational aspects of patients’ lives by increasing the risk of anxiety and depression [10]. In light of this, an assessment of awareness is crucial for improving the understanding of the disease itself as well as the needs of individuals with the disease, thus improving their quality of life.

A cross-sectional study carried out in Saudi Arabia to assess public awareness of IBD reported that there are knowledge gaps regarding the types of IBD, the specific anatomical sites affected, and certain complications [11]. Previous research also showed that a higher level of public knowledge was the only factor shown to have a positive impact on decreasing public stigma, for instance through media campaigns [12].

The level of public awareness of IBD in the Al-Baha region of Saudi Arabia is still unknown. Owing to the paucity of data in this area, this study aimed to assess the level of awareness of the general population in the Al-Baha region towards IBD and the relationship between the level of awareness and socioeconomic status.

## METHODS

### Study design

We conducted a cross-sectional survey from April 2023 to June 2023 among the general population in the Al-Baha region, Saudi Arabia.

### Inclusion and exclusion criteria

We included residents of Al-Baha region aged between 18 and 80 years. We excluded individuals working in the health sector and patients with IBD.

### Sample size calculation

The sample size was calculated using Cochran’s equation, with a precision level of ±5% and a confidence level of 95%. The estimated population of the Al-Baha region was 487,108, and the calculated sample size was 384. The study enlisted 473 participants.

### Data collection

Data was collected using an online self-administered, anonymous validated questionnaire. An informed consent form was provided within the questionnaire. Section A of the questionnaire captured sociodemographic data, and section B assessed the participants’ awareness of the symptoms, signs, and complications of IBD. A pilot study was conducted on a small sample of 20 participants to test the suitability and clarity of the questionnaire and to estimate the time required for data collection.

### Sampling technique

A convenience random sampling technique was used to select 473 participants from the general population of Al-Baha region. All participants had an equal chance of being selected.

### Data analysis

After data collection, the data were coded and entered into SPSS 28 (IBM Corp) for analysis. Descriptive statistics such as frequencies, percentages, means, and s.d. were used to describe the sociodemographic characteristics of the participants and their level of awareness of IBD. The chi-squared test was used to identify any significant associations between the participants’ sociodemographic characteristics and their level of awareness of IBD.

## RESULTS

The study included a total of 473 participants aged between 18 and 80 years. The majority of participants (33.4%) were in the 36–45 years age group. In addition, 64.9% of participants were female (with a female-to-male ratio of 1.85:1), 77.6% were married, 69.5% had a higher education, 51.6% were employed, and 52.8% had an average monthly income between 5,000 and 15,000 Saudi Arabian Riyal (SAR). Most participants reported being residents of the following regions: Al-Baha (28.1%), Baljurashi (25.1%), and Al-Aqiq (22.8%) (Table 1).

**Table 1:** Demographic variables of the participants (*n* = 473)

		*n*	%	
**Sex**	Male	166	35.1%	
	Female	307	64.9%	
**Age (years)**	18–25	86	18.2%	
	26–35	66	14.0%	
	36–45	158	33.4%	
	46–55	105	22.2%	
	>55	58	12.3%	
**Marital status**	Single	91	19.2%	
	Married	367	77.6%	
	Other	15	3.1%	
**Education**	Primary school	8	1.7%	
	Intermediate or high school	82	17.3%	
	Diploma	54	11.4%	
	Higher education	329	69.5%	
**Occupational status**	Unemployed	108		22.8%
	Employee	244	51.6%	
	Retired	71	15.0%	
	Other	50	10.6%	
**Monthly income**	<5,000 SAR	138	30.5%	
	5,000–10,000 SAR	109	24.1%	
	10,000–15,000 SAR	130	28.7%	
	>15,000 SAR	76	16.8%	
**Region**	Al-Aqiq	108	22.8%	
	Al-Baha	133	28.1%	
	Baljurashi	119	25.1%	
	Al-Makhwah	41	8.6%	
	Al-Qura	33	6.9%	
	Al-Mandaq	16	3.3%	
	Other	3	0.6%	

Almost two-thirds of the participants (61.7%) had never heard of or read about IBD. Social media was the most prevalent source of information for individuals who had heard of IBD (40.6%). More than two-thirds of the participants (68.4%) stated that no one in their immediate circle had been diagnosed with IBD, and 74.0% were unaware that there are different types of IBD (Table 2).

**Table 2:** General awareness level of the participants of IBD (*n* = 473)

		*n*	%
**Have you ever heard of or read about IBD?**	No	292	61.7%
	Yes	133	28.1%
	I do not remember	48	10.1%
**If the answer is yes, what is the source of your information about IBD?**	Social media	54	40.6%
	Health care specialist	13	9.8%
	Friend/family member	30	22.6%
	Awareness campaigns	19	14.3%
	Discussion in the workplace	3	2.2%
	I do not remember	14	10.5%
**Has anyone in your close circle been diagnosed with IBD?**	No	323	68.4%
	Yes	51	10.8%
	I do not know	98	20.8%
**How many types of IBD do you know?**	I do not know	348	74.0%
	1	31	6.6%
	2	46	9.8%
	3	23	4.9%
	More than 3	22	4.7%
**Have you ever heard of CD?**	No	363	77.4%
	Yes	106	22.6%
	I do not remember	0	0.0%
**CD affects:**	I do not know	304	64.3%
	Gastrointestinal system	147	31.1%
	Lung system	17	3.6%
	Liver	5	1.1%
**Have you ever heard of UC?**	No	176	37.4%
	Yes	295	62.6%
**UC affects:**	I do not know	154	32.6%
	Intestine	317	67.0%
	Kidney	2	0.4%

**Figure 1:**
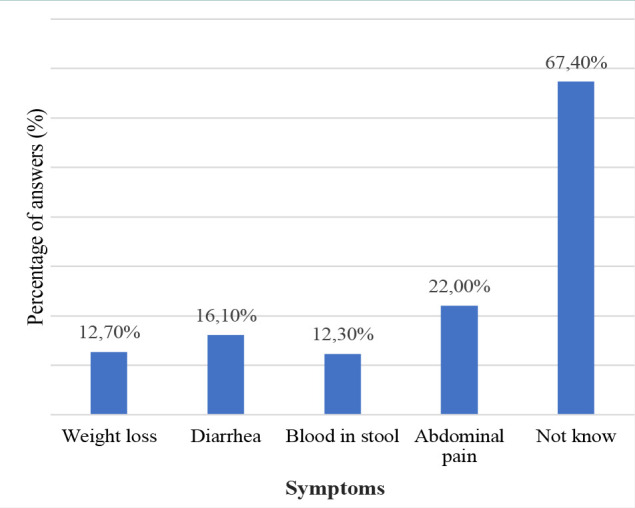
The awareness of participants regarding the symptoms of CD

**Figure 2:**
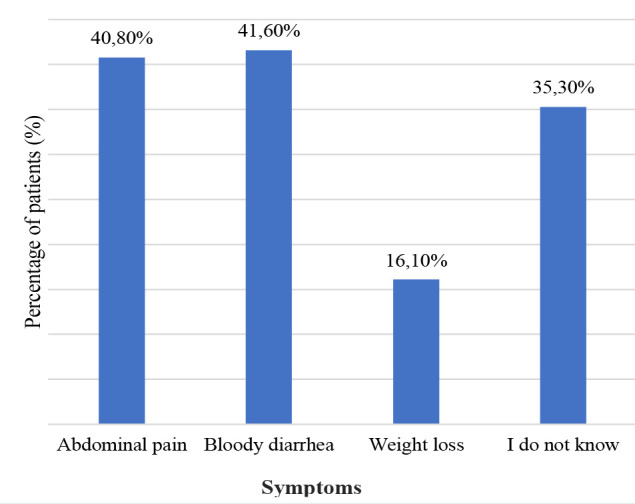
The awareness of participants regarding the symptoms of UC

The most frequently reported known symptoms of Crohn’s disease (CD) were abdominal discomfort (22.0%) and diarrhea (16.1%) (Figure 1). On the other hand, the most frequently reported known symptoms of ulcerative colitis (UC) were bloody diarrhea (41.6%) and abdominal pain (40.8%) (Figure 2). Altogether, 67.4% of participants did not know the symptoms of CD, and 35.30% were unaware of the symptoms of UC. In addition, 49.3% were aware that IBD is not contagious, 51.8% were not aware that IBD can have extraintestinal effects, and 51% did not know about the increased risk of colorectal cancer among patients with IBD compared to the general population (Table 3).

**Table 3:** Assessment of the knowledge of the participants about different factors for IBD

	No		Yes		I do not know	
	*n*	%	*n*	%	*n*	%
**Do you think that IBD is contagious?**	233	49.3%	61	12.9%	179	37.8%
**Do you think that there can be symptoms in IBD that are not directly related to the digestive system?**	89	18.8%	139	29.4%	245	51.8%
**Do you think that IBD may cause joint pain?**	87	18.4%	141	29.8%	245	51.8%
**Do you think that IBD may cause pain or redness in the eye?**	100	21.1%	98	20.7%	275	58.1%
**Can fractures be a long-term complication of IBD?**	104	22.0%	56	11.8%	313	66.2%
**Could colorectal cancer be a long-term complication of IBD?**	10	2.1%	241	51.0%	222	46.9%
**Do you think that it is possible that IBD causes blood clotting in the veins?**	37	7.9%	99	21.1%	333	71.0%
**Do you think that IBD is closely associated with liver disease?**	35	7.4%	127	27.0%	308	65.5%
**Do you think that a patient with IBD can also suffer from the risk of developing kidney stones?**	52	11.0%	117	24.8%	302	64.1%
**Do you think that IBD causes intestinal obstruction?**	26	5.5%	214	45.2%	233	49.3%
**Do you think that the risk of IBD increases with age?**	45	9.6%	198	42.0%	228	48.4%
**Do you think that it is possible to prevent the development of IBD?**	21	4.5%	254	53.9%	196	41.6%
**Do you think that IBD can be completely cured once a person is infected?**	67	14.3%	163	34.8%	239	51.0%
**Do you think that there is enough awareness in your community about IBD?**	305	65.0%	43	9.2%	121	25.8%

**Figure 3:**
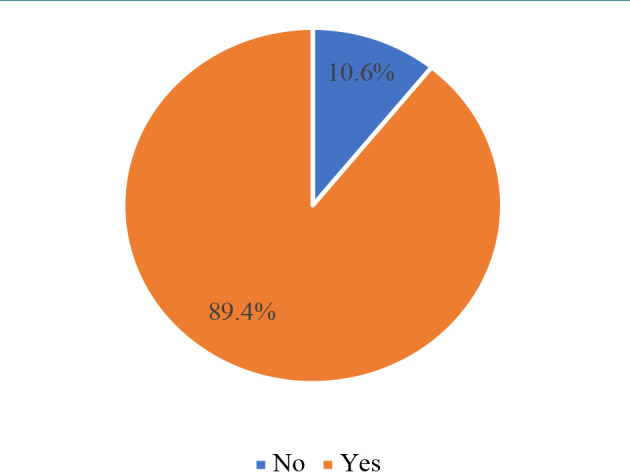
Distribution of answers to the question “Do you suggest to improve public awareness about IBD through educational campaigns or sessions?”

**Figure 4:**
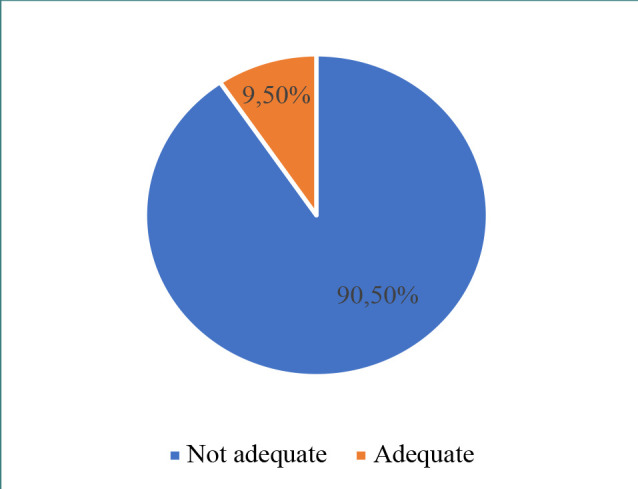
Distribution of the level of knowledge among participants

Most participants (89.4%) agreed that educational campaigns or sessions are necessary for increasing public knowledge on IBD (Figure 3). However, 90.5% demonstrated poor understanding of IBD, whereas only 9.5% exhibited adequate knowledge, correctly answering at least 60% of the questions (Figure 4).

The proportion of women who had an adequate level of knowledge was significantly higher compared to men (12.1% vs. 4.8%, *P* = 0.011). However, there was no significant association between the level of knowledge, age, marital status, occupation, or monthly income (*P* > 0.05). Regarding educational level, there was a trend toward a higher level of knowledge among participants with a higher level of education, although it was not statistically significant (*P* = 0.169) (Table 4).

**Table 4:** The association between the level of knowledge and demographic factors

		Knowledge				
		Not adequate		Adequate		*P* value
		*n*	%	*n*	%	
**Sex**	Male	158	95.2%	8	4.8%	0.011
	Female	270	87.9%	37	12.1%	
**Age (years)**	18–25	73	84.9%	13	15.1%	0.189
	26–35	59	89.4%	7	10.6%	
	36–45	143	90.5%	15	9.5%	
	46–55	97	92.4%	8	7.6%	
	>55	56	96.6%	2	3.4%	
**Marital status**	Single	81	89.0%	10	11.0%	0.180
	Married	336	91.6%	31	8.4%	
	Other	11	73.3%	4	26.7%	
**Education**	Primary school	8	100.0%	0	0.0%	0.169
	Intermediate or high school	78	95.1%	4	4.9%	
	Diploma	46	85.2%	8	14.8%	
	Bachelor’s degree	271	90.6%	28	9.4%	
	PhD or master’s degrees	25	83.3%	5	16.7%	
**Occupational status**	Unemployed	97	89.8%	11	10.2%	0.896
	Employee	220	90.2%	24	9.8%	
	Retired	66	93.0%	5	7.0%	
	Other	45	90.0%	5	10.0%	
**Monthly income**	<5,000 SAR	121	87.7%	17	12.3%	0.307
	5,000–10,000 SAR	101	92.7%	8	7.3%	
	10,000–15,000 SAR	119	91.5%	11	8.5%	
	>15,000 SAR	72	94.7%	4	5.3%	

## DISCUSSION

IBD affects millions of individuals globally [13,14]. The importance of raising public awareness about IBD is demonstrated by the fact that it significantly lowers quality of life and may lead to permanent disability [15]. In addition, despite the prevalence of IBD, there is a lack of understanding and awareness among the general population, which places a heavy burden on patients, their families, and healthcare systems [15]. Given that the frequency of IBD is increasing globally and in Saudi Arabia, it is crucial to recognize and diagnose the condition at an early stage to ensure appropriate management and to avoid complications. In this context, it is essential to assess the general public’s awareness of IBD and to identify knowledge gaps that may hinder the implementation of an adequate educational plan.

Two-thirds of the participants had neither heard of nor read about IBD, according to the findings of our study. Social media was the most popular source of information among individuals who had heard about IBD. Most participants were uninformed about the two types of IBD, and only a minority were familiar with CD and which organs it affects. Similarly, many individuals were unaware which organs does UC affect. Bloody diarrhea and abdominal pain were the most reported known symptoms of UC. Alharbi *et al*. found that the knowledge level of 200 primary care physicians in the western area of Saudi Arabia was low; however, education on IBD greatly increased their awareness [16].

The awareness of the general population of IBD has not been adequately studied [17]; we found only a few studies that evaluated awareness of IBD in Saudi Arabia. A previous study conducted in different regions of Saudi Arabia reported that individuals from both the eastern (63.1%) and western (65.1%) regions had poor knowledge of IBD [18]. Another study revealed that 40% and 36% of the sample population had never heard of or did not know about CD and UC, respectively, but only about a third did not know what organs are affected by CD and UC [17]. A study conducted in Al-Baha region reported that 33.8% of the participants responded affirmatively when asked if they had heard of IBD [19]. Another study conducted in Taif region found that 5.5% of the participants did not have any knowledge about IBD, 42.7% had a low level of knowledge, and 9% had a high level of knowledge of IBD [20]. However, a study conducted in Australia found higher knowledge scores among 409 primary healthcare physicians even without IBD-specific education [21]. Moreover, when asked 12 questions about the etiology, symptoms, and treatment of IBD, only 55% responded correctly, whereas 86% responded incorrectly to eight of the 12 questions. Statistically, however, individuals with a higher level of education performed better [12]. A nationwide survey by Angelberger *et al*. investigated the public’s awareness of IBD among 1,001 Austrians and determined that the population’s understanding was inadequate [22]. The study showed that 69% and 80% of participants had not heard of or were unaware of CD and UC, respectively. Similarly, 64% and 73% did not know which organs are affected by CD and UC, respectively [22]. Our results show that less than 20% of the participants possessed adequate knowledge, which is much lower than the 58% reported in a previous study conducted in Saudi Arabia [11]. Similar outcomes were found in a study conducted in the western part of Saudi Arabia [17].

Our study also revealed a lack of knowledge regarding the long-term effects of IBD. For example, only a minority of the participants were aware that IBD can cause fractures (11.8%), venous thromboembolism (21.1%), or be directly linked to liver diseases (27.0%). In addition, 51.0% of the participants did not know that IBD can be treated, and 65.0% did not know whether there was sufficient awareness of IBD in their community. These results are worse than those from previous studies conducted among the Saudi Arabian population, in which 60% of the participants agreed that IBD causes hypercoagulability and leads to venous thrombosis, and 65.6% were aware that pathological fractures, colorectal cancer, and kidney stones are among the long-term complications of IBD [11]. However, our results are comparable to those from a Canadian study that found a low awareness of venous thrombosis as a consequence of IBD [23].

We found a strong statistical correlation between knowledge and sex (*P* = 0.011). However, there was no association between the level of knowledge and other demographic characteristics such as age, marital status, educational level, occupation, and monthly income. In a number of earlier studies, there was a considerable difference between sexes, with women achieving significantly higher average scores than men [17,24], which is consistent with our results. A previous study showed that a higher level of education has a strong association with better knowledge about IBD [18], in contrast with our results, which showed no significant differences between the participants’ educational levels.

## CONCLUSION

We found a severe lack of information and understanding about IBD among the general population in the Al-Baha region, Saudi Arabia, notably regarding the different types of IBD, their symptoms, long-term implications, and potential therapies. The participants’ sex was the only demographic parameter with a significant effect on the level of knowledge. The findings show that further educational initiatives and resources are required to promote public awareness and comprehension of IBD and to lower the burden of IBD on individuals and on healthcare systems.

## References

[1] McDowell C, Farooq U, Haseeb M (2023). Inflammatory Bowel Disease. StatPearls [Internet]. https://www.ncbi.nlm.nih.gov/books/NBK470312.

[2] Ye Y, Pang Z, Chen W (2015). The epidemiology and risk factors of inflammatory bowel disease. Int J Clin Exp Med.

[3] Abdulla M, Al Saeed M, Fardan RH (2017). Inflammatory bowel disease in Bahrain: single-center experience. Clin Exp Gastroenterol.

[4] Butt MT, Bener A, Al-Kaabi S (2005). Clinical characteristics of Crohn's disease in Qatar. Saudi Med J.

[5] Meeralam YK, Al Zanabgi A, Mosli M, Qari Y, Al Saedi M, Tashkhandi A (2023). A Regional Survey of Awareness of Inflammatory Bowel Disease among the Saudi Population. Inflamm Intest Dis.

[6] Alqahtani RM, Alghanemi A, Aljifri AM, Ghulman IM, Ashram SY, Alghamdi EA (2023). Public Knowledge of Inflammatory Bowel Diseases in Saudi Arabia: A Cross-Sectional Survey Study. Cureus.

[7] Al Fadda M, Peedikayil MC, Kagevi I (2012). Inflammatory bowel disease in Saudi Arabia: a hospital-based clinical study of 312 patients. Ann Saudi Med.

[8] Khawaja AQ, Sawan AS (2009). Inflammatory bowel disease in the Western Saudi Arabia. Saudi Med J.

[9] Taft TH, Keefer L, Leonhard C (2009). Impact of perceived stigma on inflammatory bowel disease patient outcomes. Inflamm Bowel Dis.

[10] Janicke DM, Gray WN, Kahhan NA (2009). Brief report: the association between peer victimization, prosocial support, and treatment adherence in children and adolescents with Inflammatory Bowel Disease. J Pediatr Psychol.

[11] Eid SM, Mohammad SM, Noorelahi SK (2021). Public awareness toward inflammatory bowel disease in Saudi Arabia. Int J Med Dev Ctries (online).

[12] Groshek J, Basil M, Guo L (2017). Media consumption and creation in attitudes toward and knowledge of inflammatory bowel disease: web-based survey. J Med Internet Res.

[13] M’Koma AE (2013). Inflammatory bowel disease: an expanding global health problem. Clin Med Insights Gastroenterol.

[14] Alatab S, Sepanlou SG, Ikuta K (2020). The global, regional, and national burden of inflammatory bowel disease in 195 countries and territories 1990 –2017: a systematic analysis for the Global Burden of Disease Study 2017. Lancet Gastroenterol Hepatol.

[15] Kaibullayeva J, Ualiyeva A, Oshibayeva A (2020). Prevalence and patient awareness of inflammatory bowel disease in Kazakhstan: a cross-sectional study. Intest Res.

[16] Alharbi R, Almahmudi F, Makhdoom Y (2019). Knowledge and attitudes of primary healthcare physicians toward the diagnosis and management of inflammatory bowel disease following an educational intervention: a comparative analysis. Saudi J Gastroenterol.

[17] Meeralam YK, Al Zanabgi A, Mosli M, Qari Y, Al Saedi M, Tashkhandi A (2023). A regional survey of awareness of inflammatory bowel disease among the Saudi population. Inflamm Intest Dis.

[18] Aldakhil MF, Alfentokh OK, Alfarah MM (2022). Prevalence and knowledge about inflammatory bowel diseases in Saudi society. Int Res J Public Environ Heal.

[19] Alghamdi AG, Almuhanna ZJA, Bu Hulayqah ZM, Algharsan FAG, Alghamdi HA, Alzahrani HMA (2022). Public awareness of colorectal cancer screening in the Al-Baha region, Saudi Arabia, 2022. Cureus.

[20] Mahfouz ME, Alotaibi FH, Althumali NK, Althobaiti KT, Alqarni MM, Almnjwami RF (2022). Assessment of inflammatory bowel diseases among Taif community in Saudi Arabia. Int J Med Dev Ctries.

[21] Tan M, Holloway RH, Lange K, Andrews JM (2012). General practitioners’ knowledge of and attitudes to inflammatory bowel disease. Intern Med J.

[22] Angelberger S, Vogelsang H, Novacek G (2009). Public awareness of Crohn’s disease and ulcerative colitis: a national survey. J Crohns Colitis.

[23] Huang V, Mishra R, Thanabalan R, Nguyen GC (2013). Patient awareness of extraintestinal manifestations of inflammatory bowel disease. J Crohns Colitis.

[24] Knudsen MD, Hoff G, Tidemann-Andersen I, Bodin GE, Øvervold S, Berstad P (2021). Public awareness and perceptions of colorectal cancer prevention: a cross-sectional survey. J Cancer Educ.

